# Inhibitory effect of ginsenoside Rg3 on cancer stemness and mesenchymal transition in breast cancer via regulation of myeloid-derived suppressor cells

**DOI:** 10.1371/journal.pone.0240533

**Published:** 2020-10-22

**Authors:** Joong-Hyun Song, Da-Young Eum, Soon-Yong Park, Yun-Ho Jin, Jae-Woong Shim, Shin-Ji Park, Min-Young Kim, Seong-Jun Park, Kyu Heo, Yoo-Jin Choi

**Affiliations:** Department of Research Center, Dongnam Institute of Radiological & Medical Sciences, Busan, Republic of Korea; Sechenov First Medical University, RUSSIAN FEDERATION

## Abstract

Ginsenoside Rg3 (Rg3) has been studied in several cancer models and is suggested to act through various pharmacological effects. We investigated the anticancer properties of Rg3 through myeloid-derived suppressor cell (MDSC) modulation in FM3A mouse mammary carcinoma cells. The effects of Rg3 on MDSCs and consequent changes in cancer stem-like cells (CSCs) and epithelial-mesenchymal transition (EMT) were evaluated by diverse methods. MDSCs promoted cancer by enhancing breast cancer stemness and promoting EMT. Rg3 at a dose without obvious cytotoxicity downregulated MDSCs and repressed MDSC-induced cancer stemness and EMT. Mechanistic investigations suggested that these inhibitory effects of Rg3 on MDSCs and corresponding cancer progression depend upon suppression of the STAT3-dependent pathway, tumor-derived cytokines, and the NOTCH signaling pathway. In a mouse model, MDSCs accelerated tumor progression, and Rg3 delayed tumor growth, which is consistent with the results of *in vitro* experiments. These results indicated that Rg3 could effectively inhibit the progression of breast cancer. The anticancer effect of Rg3 might be partially due to its downregulation of MDSCs and consequent repression of cancer stemness and EMT in breast cancer. Hence, we suggest the regulation of MDSCs through Rg3 treatment as an effective therapeutic strategy for breast cancer patients.

## Introduction

Breast cancer is one of the most commonly diagnosed female cancers worldwide, and it is estimated that approximately one-third of newly diagnosed cancers in women in 2019 were breast cancer [1]. Although the breast cancer mortality rate has declined dramatically with recent advances in early diagnosis and intervention, patients with advanced breast cancer still have a poor prognosis despite comprehensive and intensive treatment [2]. Furthermore, although objective clinical responses to some chemotherapy regimens are obtainable, most advanced cancer patients suffer from the side effects of chemotherapy and resistance to chemotherapeutic agents at the end of life. Hence, there is an urgent need for the development of promising treatment options to reduce drug toxicity and resistance, restrict tumor progression, improve quality of life, and prolong survival.

Cancer-mediated immunosuppression and evasion of the immune response in the tumor microenvironment (TME) are important contributors to the malignant progression of cancers and emerging obstacles to cancer immunotherapy in various types of malignant tumors [3]. Myeloid-derived suppressor cells (MDSCs) are a heterogeneous population of myeloid cells in the early stages of differentiation that expand in various pathological conditions, including malignant tumors and chronic inflammation. Their normal maturation is halted at the defective myeloid cell stage, resulting in the eventual accumulation of MDSCs and deleterious biological effects. MDSCs mediate tumor-induced immunosuppressive activities and are directly implicated in the promotion of tumor angiogenesis, invasion, and metastasis [4]. The major immunosuppressive mechanisms of MDSCs include the production of arginase-1 (ARG1); inducible nitric oxide synthase (iNOS); and reactive oxygen species (ROS), which are implicated in T-cell dysfunction and apoptosis [5]. In addition, MDSCs secrete immunosuppressive cytokines that contribute to the induction of regulatory T cells, such as TGF-β and IL-10 [6]. MDSCs also amplify immunosuppressive activities in the TME through their interaction with other components of the immune system, such as macrophages and dendritic cells [7]. Mouse MDSCs express the myeloid lineage marker CD11b and the granulocytic marker Gr1 [4]. Gr1 includes two distinct Ly6C and Ly6G epitopes, and MDSCs are further divided into monocytic MDSCs (M-MDSCs) and granulocytic polymorphonuclear MDSCs (PMN-MDSCs) based on their expression of Ly6C and Ly6G, respectively [8]. Recently, similar to that in other malignancies, the expansion of MDSCs in the circulation or tumor tissues of breast cancer patients has been actively investigated [9, 10]. These previous studies suggested that MDSC levels correlate with tumor burden and that modulation of MDSC-tumor interactions is a promising strategy for the treatment of breast cancer.

Cancer cell stemness is a major feature of malignant cancer cells and describes the overall properties of cancer stem-like cells (CSCs), such as their self-renewal and multilineage differentiation [11]. These stem-like behaviors of CSCs play a key role in tumor initiation, metastasis and progression and therapeutic resistance. Epithelial-mesenchymal transition (EMT) is an important physiological process during embryogenesis, wound healing, and tissue regeneration whereby epithelial cells are transformed into mesenchymal cells. For epithelial malignancies, EMT is a crucial step toward cancer migration, invasion and metastasis [12]. Furthermore, the reciprocal relationship between EMT and CSCs was recently highlighted. The metastatic potential of CSCs is driven by EMT through the expression of regulatory transcription factors, and EMT also induces stem cell properties in non-stem cancer cells [13]. Thus, as both stemness and EMT are deeply implicated in the pathogenesis and development of malignant cancer, several protein markers to identify cancer stemness and EMT have been widely used to quantify the functional status of cancer cells.

Ginseng, the root of *Panax ginseng* Meyer, is popular around the world as a traditional medicine, and a number of studies on ginseng and its extracts have documented its wide range of medical applications. The role of ginsenoside Rg3 (Rg3), the main active component extracted from the natural product ginseng, in several types of cancer has already been studied, and the pharmacological effects of Rg3 are suggested to be responsible for its actions. For instance, treatment with Rg3 was shown to have significant anticancer activity against breast, colorectal, lung, ovarian, and prostate cancers [14–18]. In addition to the anticancer activities of Rg3, the safety of Rg3 in human patients and its protective role against chemotherapeutic toxicity are supported by previous studies. Suggested mechanisms of the anticancer activity of Rg3 include its induction of apoptosis; inhibition of proliferation, metastasis, and angiogenesis; augmentation of chemosensitivity; and inhibition of chemotherapy-induced toxicity [19]. One previous study also suggested immunomodulatory action as an indirect but potent anticancer activity of Rg3 [18]. However, the exact molecular mechanisms of Rg3 in cancer remain to be clarified.

In this work, we evaluated the *in vitro* effects of Rg3 on MDSCs and breast cancer and analyzed the relationship of Rg3 and MDSCs with *in vivo* anticancer activity in tumor-bearing mice. In addition, we attempted to clarify the cellular and molecular relationships among Rg3, MDSCs, and breast cancer by multimodal investigation.

## Materials and methods

### Cell line and culture

FM3A cells, a C3H/He mouse mammary carcinoma cell line, were maintained in RPMI 1640 medium (WelGENE, Korea) supplemented with 10% fetal bovine serum (FBS) (HyClone Laboratories, UT, USA) and 1% penicillin/streptomycin (Gibco, NY, USA) at 37°C in humidified air containing 5% CO_2_. Cells were passaged every 2–3 days to obtain exponential growth.

### Reagents

Rg3 with approximately 98% purity was purchased from Sigma-Aldrich (SML0184, Sigma-Aldrich Chemical Co., MO, USA). Rg3 was dissolved in culture medium for *in vitro* experiments and saline for orogastric administration. FM3A cells and MDSCs were treated with Rg3 at different concentrations (0, 3, 6, 12.5, 25 μg/ml) for different durations (24, 48, 72 h) to establish an adequate Rg3 regimen without overt toxicity, and their cell viability was assessed. Live cells were counted using a commercial cell counter (EVE automatic cell counter, NanoEnTek, Korea).

### Animals

A murine model was established in 6-week-old female C3H/He mice (Central Lab Animal Incorporation, Korea). The animals were raised under SPF conditions according to Good Laboratory Practice (GLP) OECD guidelines. The animals were maintained in a room at 23 ± 1°C, with a relative humidity of 50 ± 10%, artificial lighting from 08:00 to 20:00, and 14–18 air changes per hour. The mice were fed a standard animal diet and water ad libitum. This animal experiment was performed with the approval of the Ethics Committee (approval No. Dongnam Institute of Radiological & Medical Sciences animal ethical committee-2019-005) for the appropriateness of the method to confirm the anti-cancer efficacy of Rg3 *in vivo*, not a standardized test method. FM3A cells were cocultured with MDSCs in the presence of Rg3 (12 μg/ml) for 3 days, and FM3A cells (2×10^6^ cells/50 μl) suspended in phosphate-buffered saline (PBS) were inoculated subcutaneously into the right flanks of the C3He mice. The mice were randomly divided into control, MDSC and MDSC + Rg3 treatment groups. After the mice were sacrificed by carbon dioxide inhalation at 21 days post-injection, the bone marrow and spleen were collected, and representative images were taken. In addition, another cohort was established to identify the *in vivo* effects of Rg3 at three different doses (2.5, 5, and 10 mg/kg). FM3A cells (2×10^6^ cells/50 μl) suspended in PBS were inoculated subcutaneously into the right flanks of the C3He mice. After tumor development (to approximately 40 mm^3^), tumor-bearing mice were randomly divided into control and RG3 treatment groups. Rg3 was administered via orogastric gavage to each mouse five times a week for 3 successive weeks. Tumor development was monitored, and tumor volume was measured two times a week after tumor inoculation using the following formula: tumor volume = (width)^2^ × length × 0.52. Humane endpoints were determined before the tumor develops into necrosis and ulcers and interferes with feeding and watering, followed the guidelines of the International Council for Laboratory Animal Science (ICLAS). Mice were sacrificed through abdominal aorta blood collection under ether anesthesia at 24 days post-injection (maximum tumor size, approximately 1000 mm^3^) and bone marrow, spleen, serum, tumor were harvested and representative images were taken.

### Flow cytometric analyses and cell sorting

Cells were separately obtained from the bone marrow and spleen. Following cell isolation, red blood cells were lysed using red blood cell lysis buffer (Hybri-Max, Sigma-Aldrich Chemical Co.). The cells were resuspended in 100 μl of a 1% FBS solution in PBS and incubated with anti-CD11b (FITC-conjugated), anti-Gr1 (APC-conjugated), anti-Ly-6G (APC-conjugated) and anti-Ly-6C (FITC-conjugated) antibodies. All antibodies used were obtained from the MACS system (Miltenyi Biotec, Germany). The cell pellets were resuspended in 400 μl of a 1% FBS solution in PBS and analyzed by flow cytometry (Navios analyzer, Beckman Coulter, Inc., FL, USA). To separate MDSCs, the cells were labeled with fluorescent antibodies against CD11b and Gr1 and sorted on a flow cytometer/sorter (FACSAria cell sorter, BD Biosciences, USA).

### Coculture

FM3A tumor cells (1×10^5^/2 ml) suspended in cell culture medium were initially plated on 6-well cell culture plates (cat. #353046, Falcon, NJ, USA). Transparent polyethylene terephthalate (PET) membrane-containing cell culture inserts with a 1.0 μm pore size (cat. #353102, Falcon) were transferred into previously filled wells, and spleen cells or MDSCs in suspension (1×10^6^/3 ml) were added to the tops of the inserts. Cells in the wells were then treated with Rg3 at different concentrations (0, 3, 6 and 12 μg/ml). After 72 h of coculture, each cells were harvested. Isolated tumor cells, spleen cells and MDSCs were washed, and flow cytometry, Western blotting, and RT-PCR were performed. The coculture supernatants were separately harvested, and ELISA, cytokine array, and NO assays were performed.

### Invasion assays

A Matrigel invasion test was performed to identify the invasiveness of FM3A cells. Transwell inserts with an 8 μm pore size (cat. #353097, Falcon) were placed into 24-well chambers and coated with Matrigel matrix (Matrigel Basement Membrane Matrix, Corning, NY, USA). FM3A cells were harvested from the coculture conditions described above, and 1×10^5^ cells were resuspended in 1 ml of serum-free medium. Then, 800 μl of culture medium was added to the outer chambers, and 150 μl of a cell suspension was added to the inner chambers. After 24 h of incubation, the inserts were washed with PBS, the invaded cells on the lower surface of the insert membrane were fixed with 100% methanol and stained with a 0.5% crystal violet solution. The invaded cells in 5 representative fields for each insert were quantified using an EVOS microscope (EVOS XL Cell Imaging System, Life Technologies) at a magnification of 200×.

### Clonogenic cell survival assay

FM3A cells cocultured with MDSCs in the presence of Rg3 at different concentrations (0, 3, 6, 12 μg/ml) were harvested and seeded into 100 mm culture plates (cat. #353003, Falcon) at 1,000 cells/plate. After 4 h, each plate was exposed to gamma rays from a ^137^Cs gamma-ray source (Eckwert & Ziegler, Germany) at a dose rate of 2.6 Gy/min. Following ionizing radiation (IR; 0, 0.5, 1, 2, 3, 4 Gy), the cells were incubated for 10 days, and colonies were fixed with methanol and stained with 0.5% crystal violet.

### Western blot assay

Protein samples were prepared from cell and tissue lysates, and protein concentrations were determined with Bradford protein assay reagent (Bio-Rad, CA, USA) using bovine serum albumin (BSA) as a standard. Samples containing equivalent amounts of total protein were separated by SDS-polyacrylamide gel electrophoresis (SDS-PAGE) and transferred onto polyvinylidene difluoride (PVDF) membranes. The membranes were blocked with 5% skim milk in TBST at room temperature (RT) for 1 h and incubated with antibodies against pSTAT3 (cat. #9145, Cell Signaling Technology, NY, USA), ALDH1 (cat. #50385, Santa Cruz Biotechnology, CA, USA), GLUT1 (cat. #7903, Santa Cruz Biotechnology), β-catenin (cat. #610154, BD Biosciences), NOTCH1 (cat. #6014, Santa Cruz Biotechnology), OCT4 (cat. #611203, BD Biosciences), KLF4 (cat. #TA324722, OriGene, MD, USA), Vimentin (cat. #550513, BD Biosciences), NOTCH2 (cat. #5732, Cell Signaling Technology), and β-Actin (cat. #47778, Santa Cruz Biotechnology). The membranes were then incubated with horseradish peroxidase-conjugated anti-rabbit and anti-mouse antibodies (cat. #G-21234 and #G-21040, respectively; Invitrogen, CA, USA) at RT for 1 h. Antibody immunostaining was achieved using ECL™ Western Blotting Detection Reagents (GE Healthcare, WI, USA), and proteins were detected using the Fusion FX5 image analyzer (Vilber Lourmat, Marne-la-VallCe, France). The quantification of immunoreactivity corresponding to the bands was also performed by densitometric analysis using Fusion-Capt Advance software (Vilber Lourmat, Marne-la-VallCe, France).

### Real-time quantitative reverse transcription-PCR

Total RNA was extracted using Ribospin (GeneAll Biotechnology, Korea) according to the manufacturer’s protocol. After the RNA concentration was quantified using a NanoDrop 2000 spectrometer (Thermo Fisher Scientific, NJ, USA), RNA was reverse transcribed into cDNA using an iScript cDNA synthesis kit (Bio-Rad Laboratories) according to the manufacturer’s protocol. Samples for real-time PCR were prepared by mixing cDNA with FastStart Essential DNA Green Master (Roche Diagnostics, Switzerland) and forward and reverse primers. Real-time PCR was performed on a LightCycler 96 system (Roche Diagnostics). Target gene expression was normalized to GAPDH. The specific primer sequences are presented in Table 1.

**Table 1:** Sequences of real-time quantitative revere transcription-PCR primers.

Target Gene	Forward primer	Reverse primer
*mGAPDH*	GAC GGC CGC ATC TTC TTG T	CAC ACC GAC CTT CAC CAT TTT
*mNotch2*	CGG ACC AGC CTG AGA ACC T	CCT CAA GAA GCT TCG CGA AT
*mNotch3*	TTG TCT GGA TGG AAG CCC ATG T	ACT GAA CTC TGG CAA ACG CCT
*mHey1*	CAC GCC ACT ATG CTC AAT GT	TCT CCC TTC ACC TCA CTG CT
*mHey2*	TTC TGT CTC TTT CGG CCA CT	TTT GTC CCA GTG CTT GTC TG

### Cytokine and nitric oxide (NO) assays

Cytokine production was measured by ELISA according to the manufacturer’s protocol (Mouse VEGF DuoSet ELISA, DY493, R&D Systems, MN, USA; Mouse G-CSF DuoSet ELISA, DY414, R&D Systems). The optical density (OD) of the samples at 450 nm was assessed using a microtiter plate spectrophotometer (Beckman Coulter). Cytokines/chemokines in the culture supernatant were analyzed using the Proteome Profiler™ mouse cytokine array kit (mouse cytokine array, panel A, ARY006, R&D Systems) according to the manufacturer’s instructions. Signals were detected using a Fusion FX5 image analyzer (Vilber Lourmat, France). NO in the culture supernatant was detected according to the manufacturer’s protocol (Total NO and Nitrate/Nitrite Parameter Assay Kit, KGE001, R&D Systems) and normalized to the medium as a control. The OD of the samples at 540 nm was assessed using a SpectraMax Paradigm microplate spectrophotometer (Molecular Devices, CA, USA).

### Immunohistochemical analysis

The excised tumor tissues were fixed in 4% buffered formalin, embedded in paraffin and sectioned. The sections were deparaffinized and rehydrated using standard protocols. Immunostaining was performed in a Leica Bond-Max automated immunostainer (Leica, Bannockburn, UK) using commercial anti-phospho-STAT3 antibody (cat. #76315, Abcam, OR, USA). The slides were incubated with 3% hydrogen peroxide in PBS for 10 min to block endogenous peroxidase. The primary antibody was applied for 20 min at RT, followed by the application of reagent from a biotin-free bond polymer refine detection kit (Bond™ Polymer Refine Detection, Leika, Bannockburn, UK) for 15 min. 3,3′-Diaminobenzidine was applied for 10 min as a chromogen to visualize the reaction. Finally, the slides were counterstained with hematoxylin, and immunohistochemical images were collected using a digital slide scanner system (Aperio ScanScope XT, Aperio Technology, IL, USA).

### Statistical analysis

The results are presented as the mean ± SD, and the significance of differences was analyzed using one-way analysis of variance (ANOVA) followed by Tukey’s or Dunnett’s test with significance defined as *P* < 0.05. The analyses were performed using GraphPad Prism 8.2.0 software (GraphPad Software, SD, USA).

## Results

### Rg3 represses tumor-induced MDSCs

**Fig 1:**
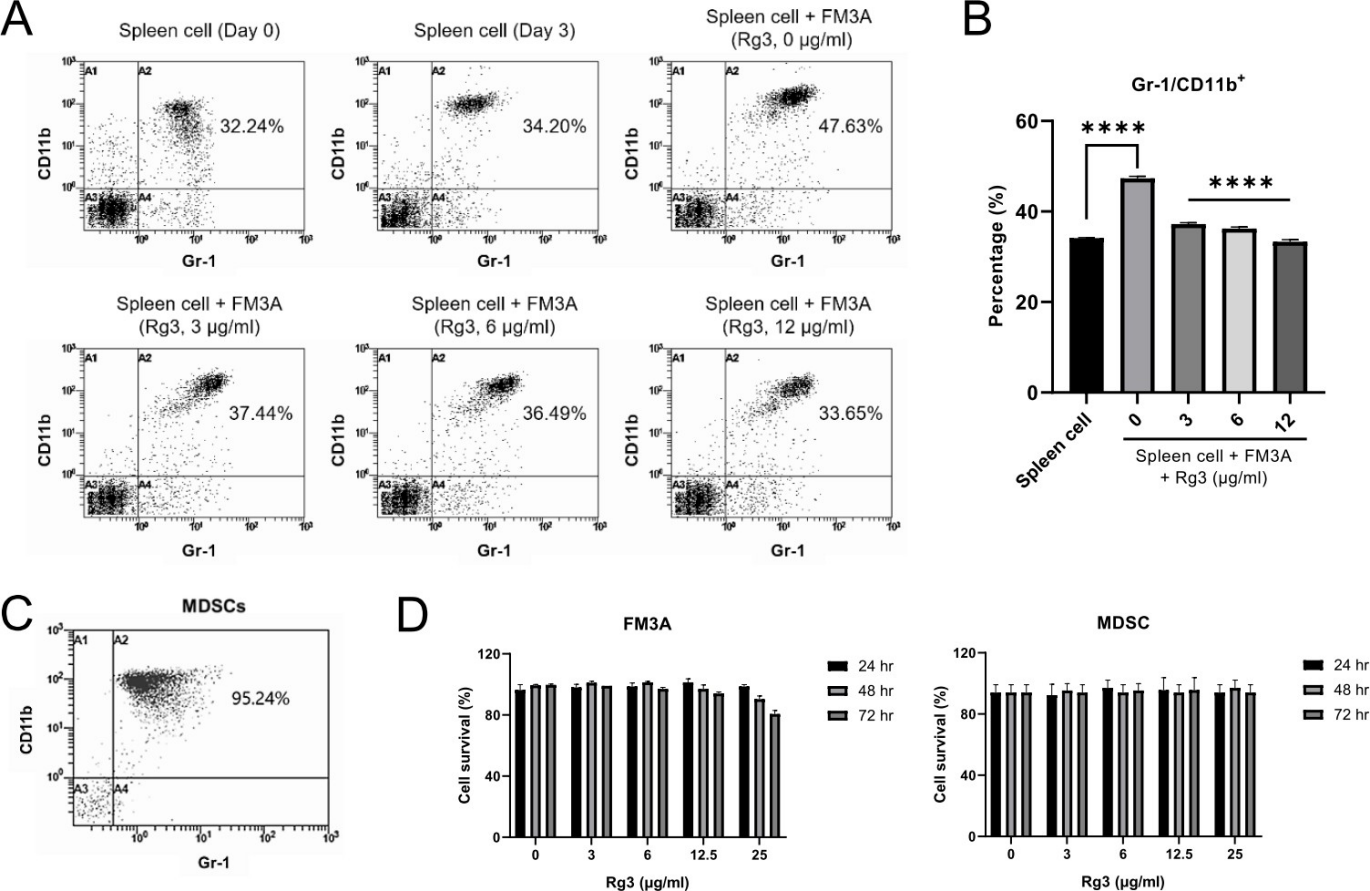
Mouse FM3A breast cancer cells induce MDSC expansion, and Rg3 represses tumor-induced MDSCs. (A) Representative flow cytometry plots and (B) graph showing the percentages of MDSCs among spleen cells under different culture conditions for 72 h. (****P < 0.0001). (C) After MDSCs were isolated from the spleens of FM3A cell tumor-bearing mice, the proportion of MDSCs was evaluated again by flow cytometry. (D and E) Cell viability tests in FM3A cells and MDSCs after treatment with Rg3 at different concentrations (0, 3, 6, 12.5, 25 μg/ml) for different durations (24, 48, 72 h) were performed to establish an adequate Rg3 regimen without overt toxicity.

To investigate the effects of Rg3 on the FM3A cell-induced expansion of MDSCs *in vitro*, the percentage of MDSCs under coculture conditions was analyzed by flow cytometry. MDSCs were expanded by coculture with FM3A cells and significantly inhibited by Rg3 treatment in a dose-dependent manner (Fig 1A and 1B). These results indicated that FM3A breast cancer cells promoted MDSCs and that Rg3 had an inhibitory effect on MDSCs.

For further experiments, we isolated MDSCs from the spleens of FM3A cell tumor-bearing mice based on their phenotype (Gr1^+^CD11b^+^) and reconfirmed the predominance of MDSCs in the sorted cell preparation (Fig 1C). A cell viability test was performed to establish an adequate Rg3 regimen, and treatment of the cells with Rg3 at 0, 3, 6, and 12.5 μg/ml resulted in no obvious cytotoxicity, while treatment with Rg3 at 25 μg/ml elicited cytotoxicity (Fig 1D). On the basis of this result, 12 μg/ml was selected as the upper limit dose in this study.

### Rg3 inhibits MDSC-induced cancer stemness and EMT

**Fig 2:**
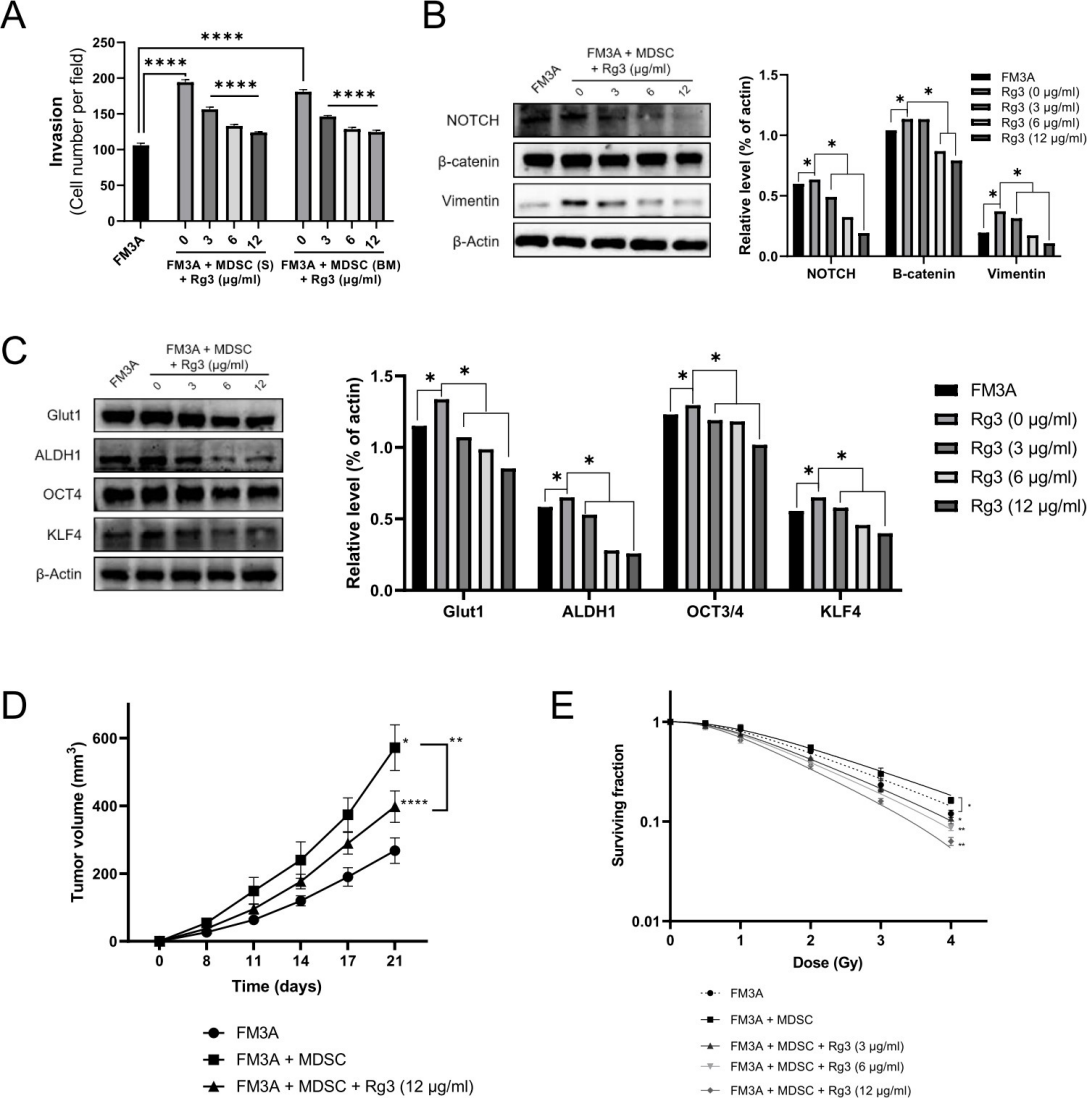
Ginsenoside Rg3 inhibits MDSC-induced cancer stemness, EMT, and tumor growth. (A) Representative graph of FM3A cell invasion efficiency after coculture with MDSCs in the presence of Rg3. Original magnification, 100×. S, spleen; BM, bone marrow. (B and C) FM3A cells and MDSCs were cocultured under the same conditions for 3 days. Expression levels of cancer stemness-related proteins and EMT-related proteins in FM3A cell lysates were detected by Western blot analysis. β-Actin was used as a control. (D) Tumor volume curves comparing the control, MDSC and MDSC + Rg3 (12 μg/ml) groups. Mice were sacrificed at 21 days post-injection. (E) Clonogenic death of FM3A cells treated with Rg3 (3, 6, 12 μg/ml) and/or irradiation (0.5, 1, 2, 3, 4 Gy) cocultured with MDSCs. *P < 0.05, **P < 0.01, and ****P < 0.0001 indicate significant differences compared with the respective control groups.

To investigate whether MDSCs could promote cancer stemness and EMT in FM3A cells and whether Rg3 could affect MDSC-induced cancer stemness and EMT, we performed *in vitro* invasion and Western blot assays. In the invasion assays, MDSCs enhanced the invasion of FM3A cells, which was significantly inhibited by Rg3 in a dose-dependent manner (Fig 2A). The Western blot assay verified that the EMT-related proteins NOTCH, β-catenin, and Vimentin were upregulated in the presence of MDSCs compared with their expression in FM3A cells alone and dose-dependently downregulated after treatment with Rg3 (Fig 2B). We further found that expression of the cancer stemness-related proteins GLUT1, ALDH1, OCT4, and KLF4 were upregulated in the presence of MDSCs and dose-dependently downregulated after treatment with Rg3 (Fig 2C). Given the importance of these *in vitro* results, an additional experiment was performed in a mouse allograft breast cancer model, and we confirmed that MDSCs accelerated tumor progression and that Rg3 repressed MDSC-mediated acceleration of tumor progression (Fig 2D). Additionally, we tested whether Rg3 affects the sensitivity of FM3A breast cancer cells to IR after their coculture with MDSCs. A clonogenic assay showed that MDSC coculture increased the resistance of FM3A cells to IR and that treatment with Rg3 reversed the radioresistance induced by MDSCs in a dose-dependent manner (Fig 2E). Altogether, these data suggest that MDSCs play an important role in promoting the cancer stemness and EMT of breast cancer, which subsequently promotes tumor progression and the resistance of breast cancer to radiation therapy. Accordingly, Rg3 treatment could impair these deleterious changes in breast cancer through inhibiting MDSCs.

### Rg3 represses MDSC-induced cytokine release

**Fig 3:**
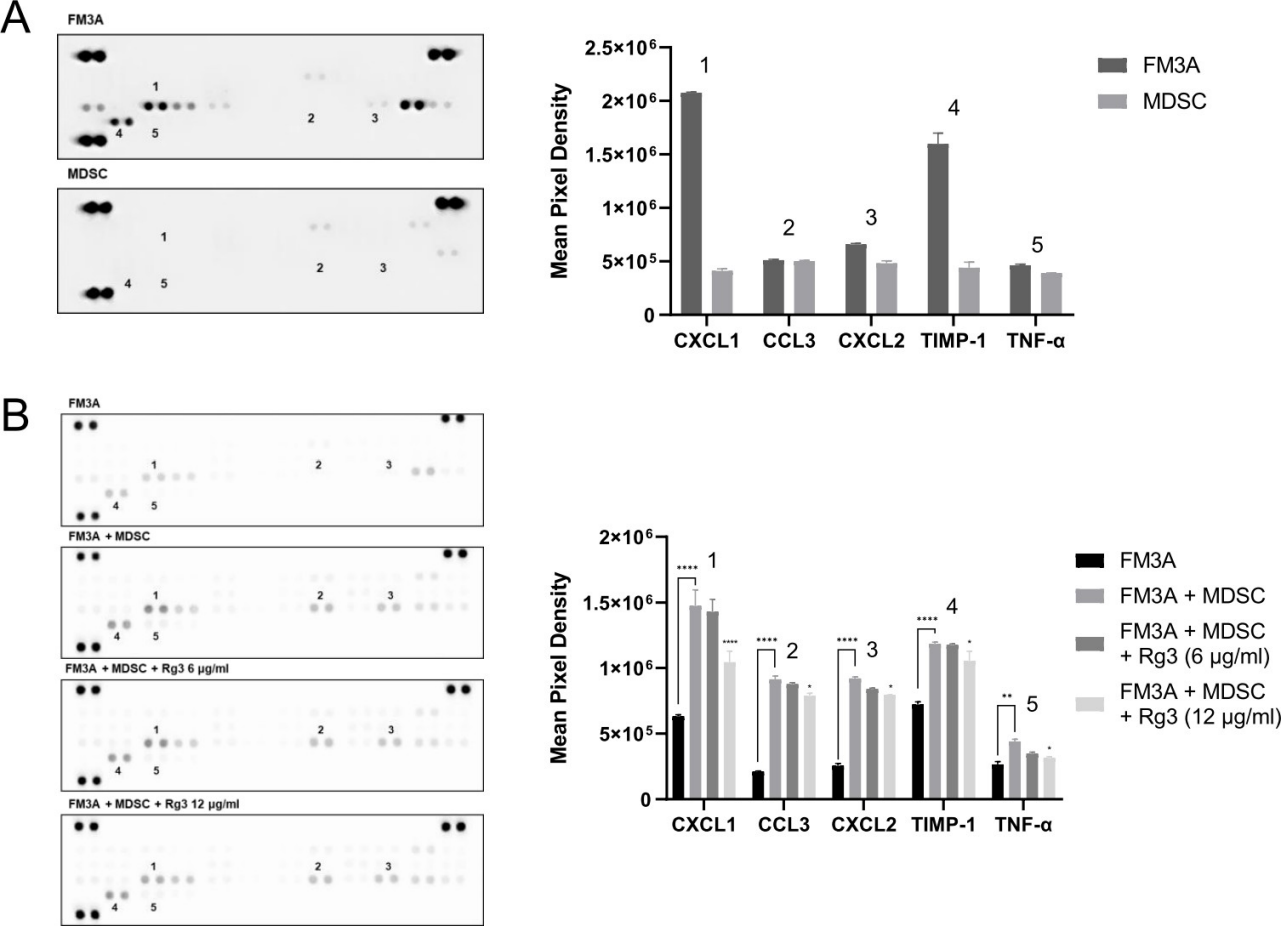
MDSCs induce the release of tumor-related cytokines/chemokines from FM3A cells, and Rg3-induced MDSC regulation represses these cytokines/chemokines. (A) The results of the cytokine array of the culture supernatants of FM3A cells and MDSCs. (B) Cytokine arrays were also performed using the coculture supernatants of FM3A cells and MDSCs grown in the presence of Rg3 at different concentrations (0, 3, 6, 12 μg/ml) for 3 days. *P < 0.05, **P < 0.01, and ****P < 0.0001 indicate significant differences compared with the respective control groups.

Given that MDSCs and Rg3 are highly correlated with the malignant behavior of FM3A cells, we examined whether and which tumor-derived cytokines from FM3A cells are involved in these biological interactions using a mouse cytokine array. MDSCs and FM3A breast cancer cells were cocultured with Rg3 at different concentrations (0, 3, 6, and 12 μg/ml), and the culture supernatants were separately harvested after 3 days of culture. We first confirmed the expression of a few cytokines in the respective cell culture supernatants of FM3A cells and MDSCs (Fig 3A). We then evaluated changes in the expression of these cytokines under different culture conditions. The expression levels of CXCL1, CCL3, CXCL2, TIMP-1, and TNF-α in the coculture supernatants of FM3A cells and MDSCs were significantly increased compared to those in FM3A cells cultured alone and further significantly decreased after treatment with Rg3 at a dose of 12 μg/ml (Fig 3B). These results suggested that MDSCs upregulated the levels of some tumor-derived cytokines and that Rg3 treatment repressed these changes in cytokine levels through downregulating MDSCs, which is consistent with decreased EMT and the downregulation of CSC markers.

### Rg3 inhibits MDSC-induced NOTCH and STAT3 signaling pathway activation

**Fig 4:**
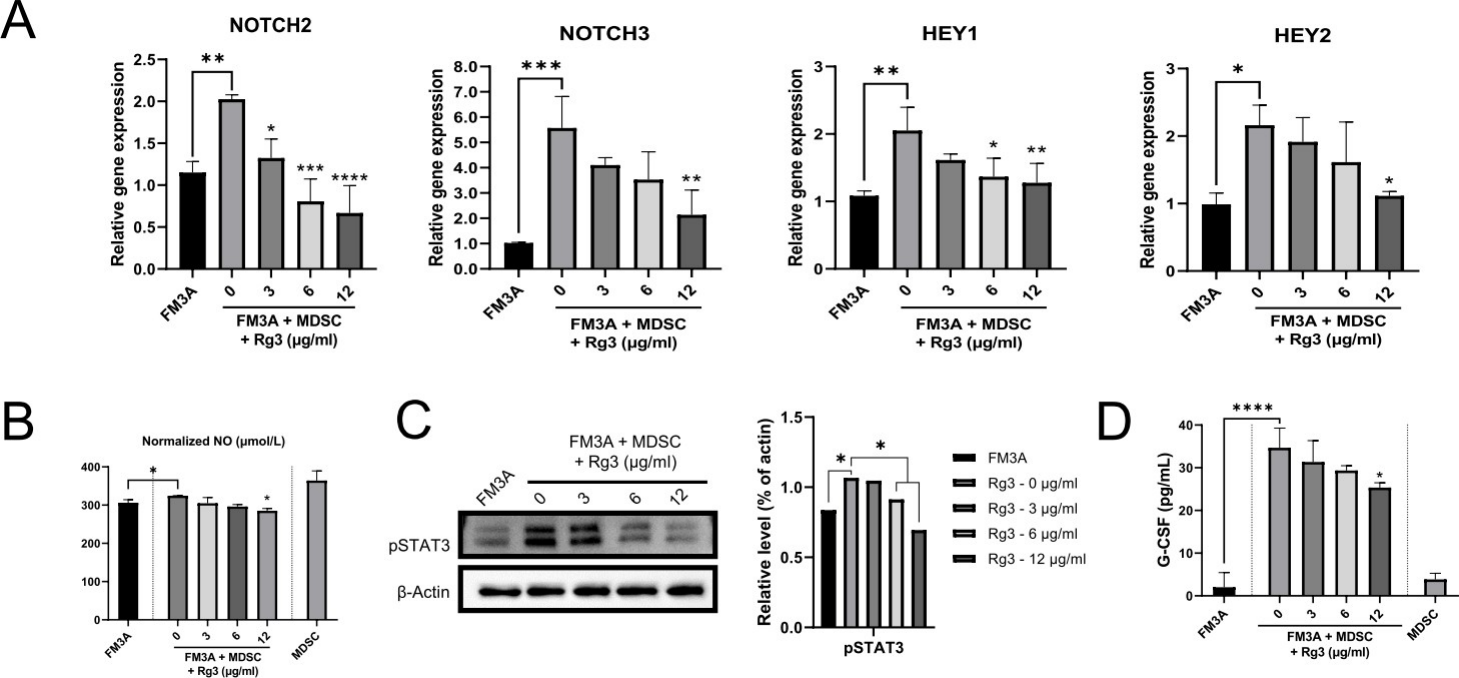
MDSCs induce NOTCH and STAT3 signaling pathway activation in FM3A cells, and Rg3 inhibits MDSC-induced NOTCH and STAT3 activation. (A) NOTCH-associated gene expression in FM3A cells cocultured with MDSCs in the presence of Rg3 at different concentrations (0, 3, 6, 12 μg/ml) for 3 days was analyzed by real-time PCR. (B) Normalized NO in cocultured cells under the same culture conditions was detected by ELISA. (C) STAT3 phosphorylation was detected in FM3A cell lysates obtained under the same culture conditions by Western blot analysis. β-Actin was used as a control. (D) Effects of Rg3 on MDSC-induced G-CSF production in FM3A breast cancer cells. The levels of G-CSF in the coculture supernatants of cells grown under the same culture conditions were detected by ELISA. *P < 0.05, **P < 0.01, ***P < 0.001, and ****P < 0.0001 indicate significant differences compared with the respective control groups (FM3A + MDSC).

Interactions between the NOTCH and STAT3 signaling pathways of MDSCs and CSCs play a crucial role in promoting and maintaining breast cancer stemness and consequent breast cancer progression [20, 21]. To investigate whether Rg3 affects activation of the NOTCH and STAT3 signaling pathways, we examined NOTCH-associated gene expression and STAT3 phosphorylation using real-time PCR and Western blot assays. Moreover, to understand the underlying mechanism, we further examined the production of NO and G-CSF, important mediators of the NOTCH and STAT3 activation cascade, by ELISA [20, 22]. As shown in Fig 4A and 4B, MDSCs significantly increased NOTCH-associated gene activation and NO production in FM3A cells. These effects were dose-dependently inhibited by treatment with Rg3. We further confirmed that MDSCs strongly induced STAT3 phosphorylation and G-CSF production in FM3A cells (Fig 4C and 4D). These effects were also significantly inhibited by treatment with Rg3. These results indicated that Rg3 treatment potently blocked MDSC-induced NOTCH and STAT3 signaling pathway activation via the downregulation of MDSCs.

### Rg3 inhibits cancer progression in an *in vivo* mouse model of breast cancer

**Fig 5:**
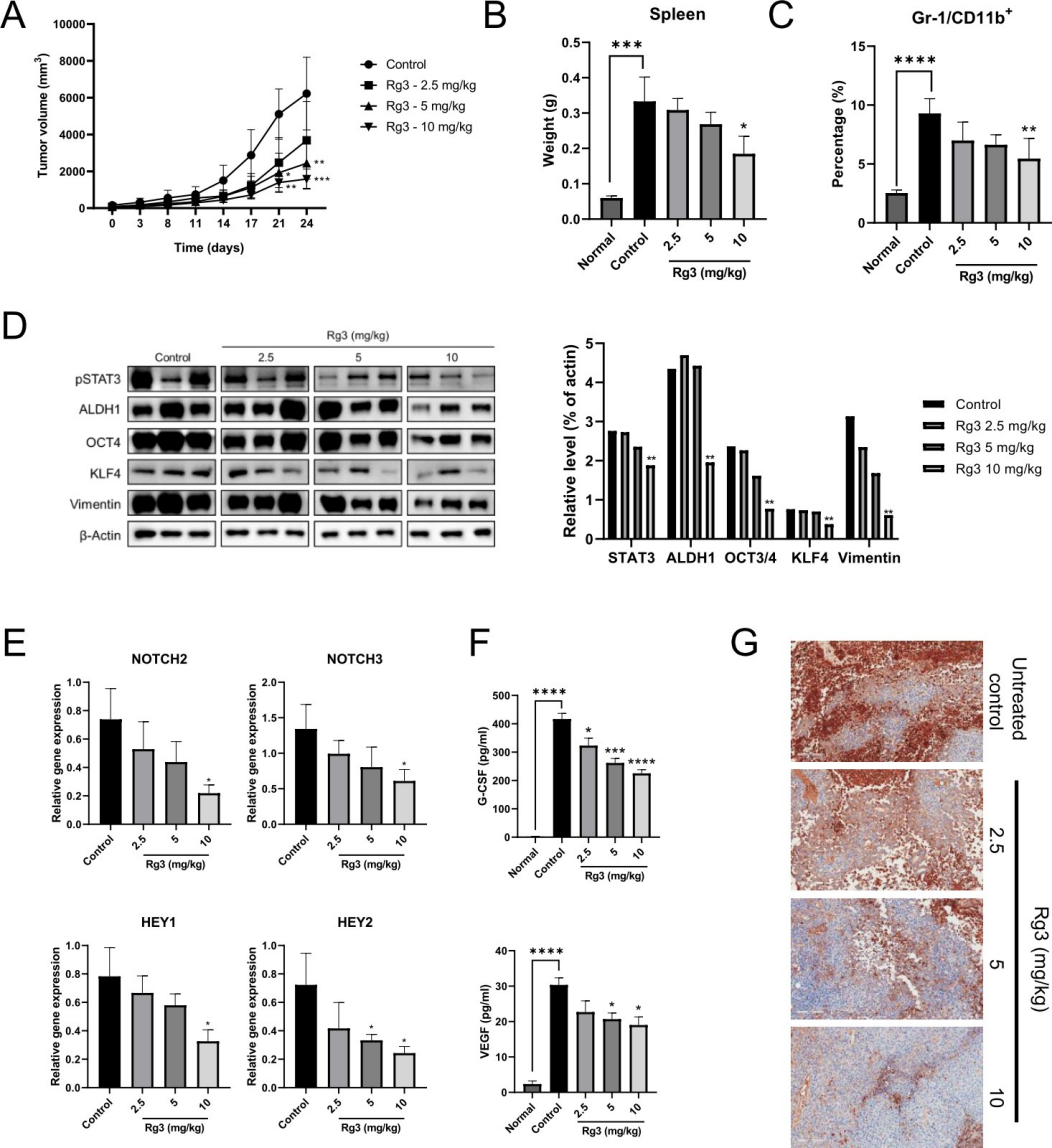
Rg3 inhibits cancer progression in an *in vivo* mouse model of breast cancer. (A) Tumor volume curves comparing the Rg3 treatment groups and the control group. (B) Spleen weight was measured on the day of sacrifice. (C) The percentage of MDSCs among spleen cells was analyzed on the day of sacrifice. (D) Western blot analysis of the expression levels of pSTAT3, ALDH1, OCT4, KLF4, and Vimentin in tumor tissues. β-Actin was used as a control. (E) NOTCH-associated gene expression in tumor tissues was analyzed by real-time PCR. (F) G-CSF and VEGF concentrations were detected with ELISA. (G) Representative immunohistochemical images showing pSTAT3 expression in tumor tissues. Original magnification, 200× (scale bar, 100 μm). *P < 0.05, **P < 0.01, ***P < 0.001, and ****P < 0.0001 indicate significant differences compared with the respective control groups.

To further identify the anticancer effects of Rg3 *in vivo*, we developed a tumor model in mice bearing FM3A breast carcinoma cell-derived tumors. As shown in Fig 5A, treatment with Rg3 for 24 days significantly repressed the growth of the tumors compared with their growth in the control group. The spleen weight on the day of sacrifice was significantly reduced after treatment with Rg3 at a dose of 10 mg/kg (Fig 5B). The percentage of MDSCs among the spleen cells was significantly reduced with Rg3 at the same dose (Fig 5C). We also found that Rg3 treatment significantly decreased the expression levels of pSTAT3, ALDH1, OCT4, KLF4, and Vimentin compared with those in the control group (Fig 5D). We then analyzed NOTCH-associated gene expression in tumor tissues and measured serum G-CSF and VEGF levels. We confirmed that Rg3 treatment at a dose of 10 mg/kg significantly decreased NOTCH-associated gene expression (Fig 5E) and the levels of G-CSF and VEGF (Fig 5F) compared with those in the control group. Immunohistochemical analysis revealed that the pSTAT3 expression level in the tumor tissues of untreated controls was significantly higher than that in the Rg3 treatment groups (Fig 5G). These results were consistent with the *in vitro* data and demonstrated the relevance of the data. Therefore, these comprehensive results verify that Rg3 has anticancer effects against mouse FM3A breast cancer.

## Discussion

The anticancer properties of ginseng have been studied in many cancer cell lines and models. Recent studies have shown that the anticancer effect of ginseng extract is closely related to cancer stemness and EMT [16, 23, 24]. A few studies have examined the effect of ginseng extract on the tumor-associated immune system. These studies suggested the role of ginseng extract in cancer as a candidate immunomodulatory agent. For example, in a previous study of thymoma-bearing mice, Korean red ginseng extract, a heat-processed extract of *Panax ginseng*, effectively inhibited the T-cell-suppressive function of MDSCs but did not decrease the number of MDSCs [25]. In another study of mouse lung cancer, the oral administration of Rg3 significantly facilitated phagocytic activity and lymphocyte proliferation [18]. Nevertheless, the mechanisms involved in these effects of ginseng extracts have not been well elucidated. Therefore, we investigated whether Rg3 can directly repress MDSCs and whether MDSCs are involved in the anticancer effect of Rg3. Moreover, we also investigated the underlying mechanisms involved in the effects of Rg3 on MDSCs.

First, we observed that MDSCs originating from the spleen were expanded by the addition of FM3A mouse breast cancer cells. Then, we identified that experimentally induced MDSCs significantly enhanced breast cancer cell invasion as well as tumor growth *in vivo*. MDSCs are a crucial player in tumor-mediated immunosuppression [4, 8, 26]. Immune dysfunction mediated by MDSCs within the TME protects cancer cells from immune attack, maintains tumor growth, and generates resistance to immunotherapy [3]. Consistent with our results, previous studies revealed that the proportion of MDSCs is closely correlated with the tumor burden, prognosis, and therapeutic response of breast cancer and other malignancies [9, 27, 28]. In addition, growing evidence has not only implicated MDSCs in antitumor immunity suppression but also suggested their directly support of cancer development and progression. Although these notions are quite difficult to clearly understand due to the complex nature of the interactions between MDSCs and cancer cells, the cancer-promoting effect of MDSCs shown by our results supports the relevance of the functions of MDSCs in breast cancer and warrants further investigation. Moreover, we suggest that the importance of MDSCs in cancer progression is more important than expected.

Several mechanisms by which MDSCs directly promote tumor progression have been proposed, and MDSCs have been suggested to inhibit apoptosis and promote proliferation [29, 30], induce and support CSCs [20, 21], enhance tumor angiogenesis [31, 32], enhance tumor invasion and metastasis [33], and produce a premetastatic environment before the arrival of tumor cells [34]. Based on these functions, a great deal of effort has been expended to clarify the link between MDSCs and CSCs in various malignancies. Cui and colleagues [28] found that MDSCs enhanced ovarian cancer stemness by inducing microRNA101 and suppressing the corepressor C-terminal-binding protein 2. Panni et al. [21] showed that M-MDSCs play an important STAT3-dependent role in promoting pancreatic cancer stemness and EMT. Peng and colleagues [20] showed that MDSCs directly promoted and maintained cancer stemness through the interaction between IL6/STAT3 and the NO/NOTCH signaling pathway. Crosstalk between STAT3 and the NOTCH pathway was shown to be closely regulated by MDSCs. Accordingly, the authors emphasized the effectiveness of the blockade of MDSCs or this interaction in the treatment of breast cancer. Consistently, we assessed changes in CSCs and EMT following modulation by experimentally induced MDSCs and eventually observed the cancer-promoting effects of MDSCs via the upregulation of cancer stemness and EMT in FM3A breast cancer cells. This finding suggests that the depletion of MDSCs can inhibit cancer progression and stemness and halt the EMT of breast cancer cells.

Along with surgery and chemotherapy, radiation therapy is used as a standard treatment option in women with breast cancer at almost every stage. Acquired resistance of cancer cells to radiation therapy impedes attainment of maximal efficacy during radiotherapy and eventually leads to poor prognosis in cancer patients. CSCs and EMT are well-known, important contributors to cancer resistance to radiation therapy [35]. In contrast, little is known about the influence of MDSCs on the radiosensitivity of breast cancer and other solid malignancies. This study found a close relationship between the cancer stemness/EMT of FM3A cells and MDSCs, suggesting that the modulation of MDSCs can be useful to adjust the radiosensitivity of breast cancer cells. In other words, our results verified that MDSCs can induce resistance to IR in FM3A cells and that this effect can be effectively blocked by Rg3. These findings provide new insight into the effectiveness of MDSC-targeted therapy for breast cancer radiosensitization. We therefore conclude that the regulation of MDSCs by Rg3 treatment not only inhibits breast cancer progression but also can assist in reducing radioresistance when combined with radiation therapy.

As expected, Rg3 effectively inhibited the expansion of MDSCs, thereby effectively suppressing cancer stemness and EMT in *in vitro* and *in vivo* experiments. Subsequently, Rg3 treatment greatly reduced breast cancer growth and radioresistance induced by MDSCs. Additionally, Rg3 treatment not only dosed-dependently inhibited tumor growth in breast cancer allograft mice but also effectively decreased the number of MDSCs in the spleens of mice in the same cohort in a dose-dependent manner. The expression levels of cancer stemness- and EMT-related proteins in cancer tissues was also dose-dependently decreased. First, these results indicate that the anticancer effects of Rg3 are highly associated with MDSC burden. Second, these findings also suggest that the anticancer effects of Rg3 depend on the dose of Rg3 administered; we thus urge future research to establish the effective dose of Rg3 in breast cancer patients.

Mechanistic investigations in the present study implicated the STAT3 and NOTCH signaling pathways in the anticancer activities of Rg3. The activation of MDSC-derived STAT3 and NOTCH, as mentioned above, is closely related to cancer stemness and consequent cancer progression [20]. In this context, these two pathways were significantly upregulated by MDSCs, and these effects were evidently reversed by Rg3 treatment in a dose-dependent manner. Furthermore, G-CSF and NO, well-recognized activation factors of the STAT3 and NOTCH signaling pathways, were also upregulated by MDSCs and downregulated by Rg3 treatment, which is consistent with prior results. In addition, the tumor-derived cytokines CXCL1, CCL3, CXCL2, TIMP-1, and TNF-α, which have been implicated in the generation, migration, and activation of MDSCs at the tumor site, were regulated by the modulation of MDSCs. These observations indicated an additional positive effect of tumor suppression by Rg3 treatment, which in turn led to the downregulation of MDSCs. Taken together, these results suggest that Rg3 can be used as an effective anticancer modality in breast cancer and that the anticancer effects of Rg3 are intimately correlated with the suppression of MDSCs. In other words, depletion of MDSCs can suppress the MDSC-related signaling pathway and consequent cancer stemness with EMT. In turn, these effects ultimately provoke anticancer activity against breast cancer. Furthermore, instead of treatment with Rg3, therapy targeting any pathway involved in these interactions could provide a good opportunity to treat and assist breast cancer patients.

In conclusion, this study demonstrated that Rg3 exerts anticancer effects by inhibiting MDSCs in FM3A mouse breast cancer cells. This anticancer effect is associated with the downregulation of cancer stemness and EMT, which could be related to the suppression of STAT3 and NOTCH signaling pathways. Therefore, targeting MDSCs is an attractive and novel approach in cancer treatment, and targeting Rg3 could be a promising therapeutic strategy in breast cancer therapy.

## Supporting information

S1 Raw images (PDF)
