# Supplementary figures and images for: Inhibitory effect of ginsenoside Rg3 on cancer stemness and mesenchymal transition in breast cancer via regulation of myeloid-derived suppressor cells
Source: PLoS One. 2020 Oct 22;15(10):e0240533. doi: 10.1371/journal.pone.0240533 (PMC7580975; doi:10.1371/journal.pone.0240533)

Figure 2B

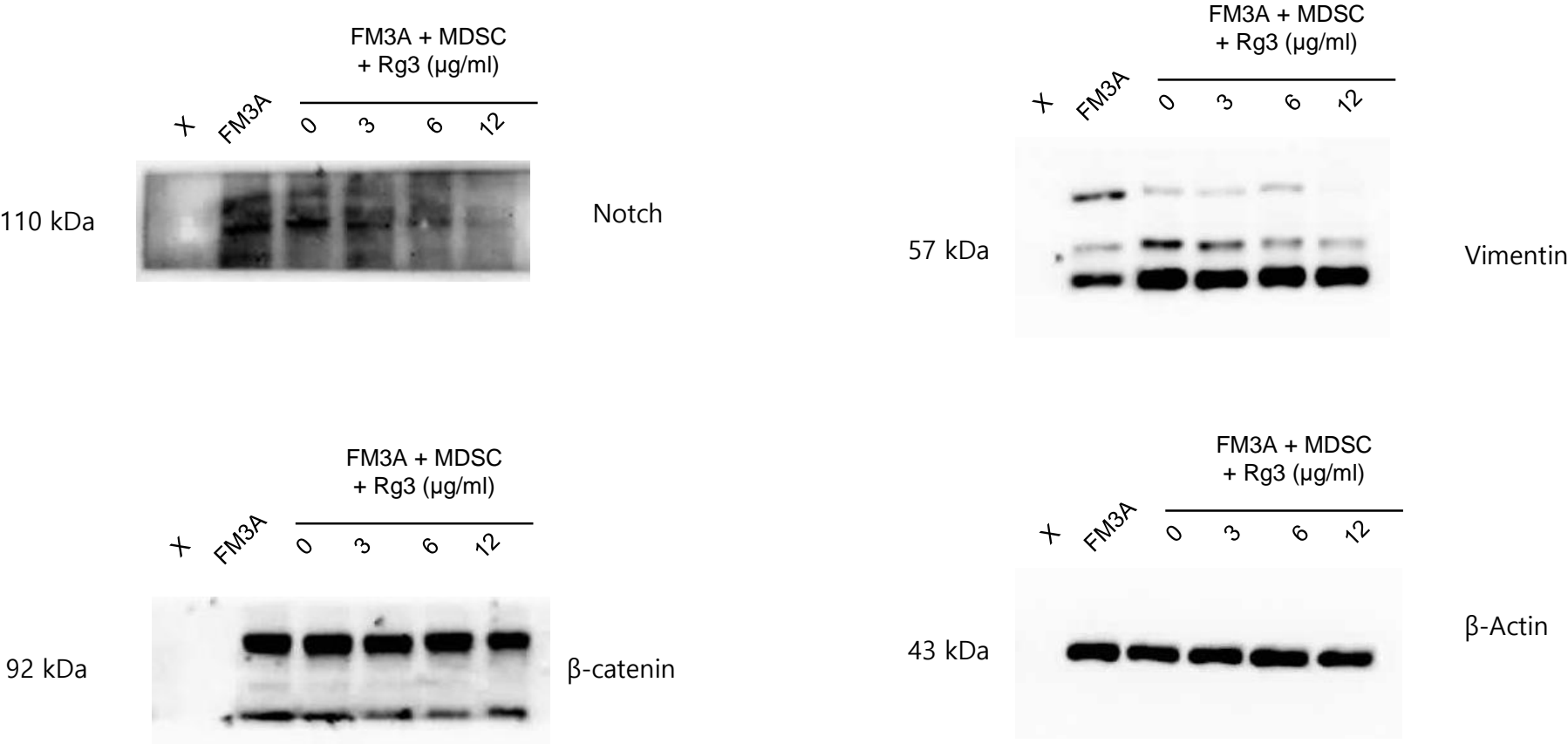

Figure 2C

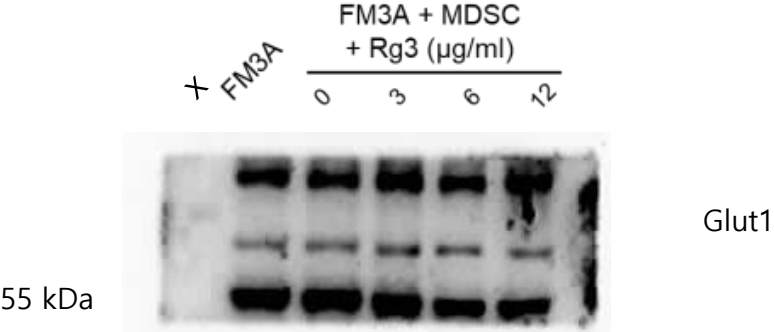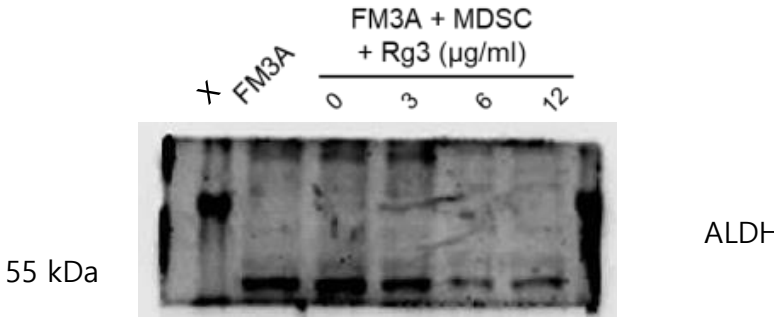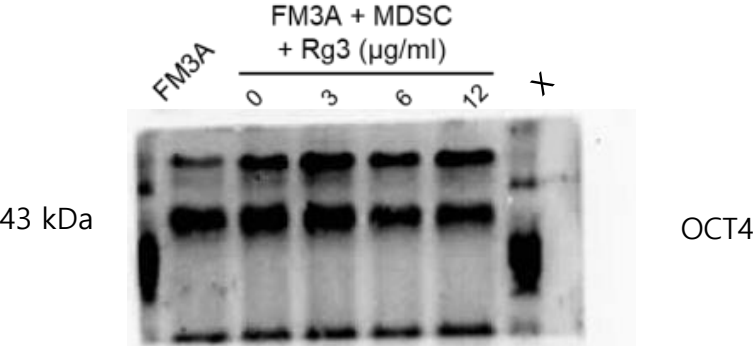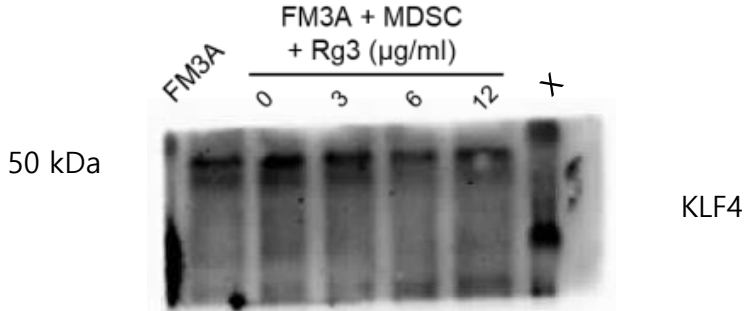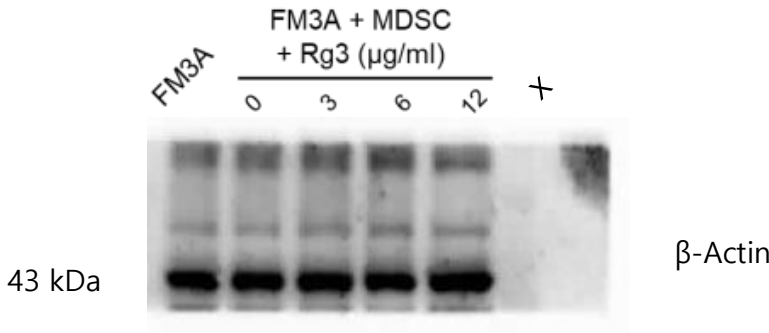

Figure 4C

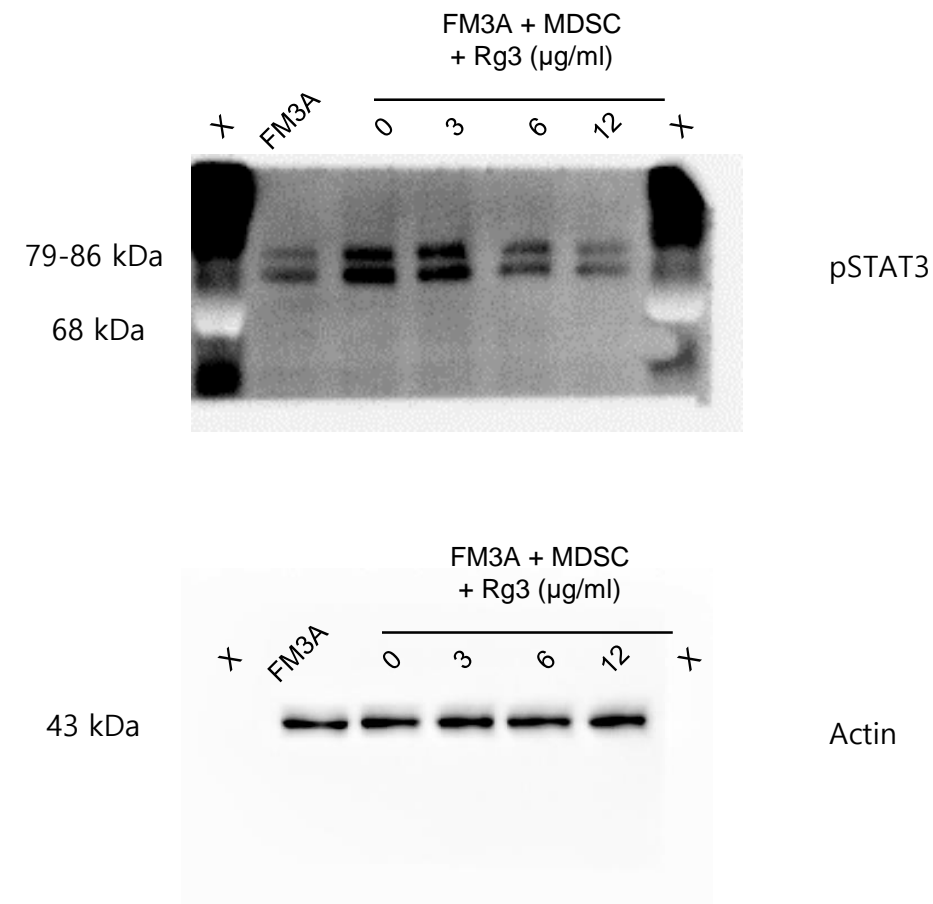

Figure 5D-1

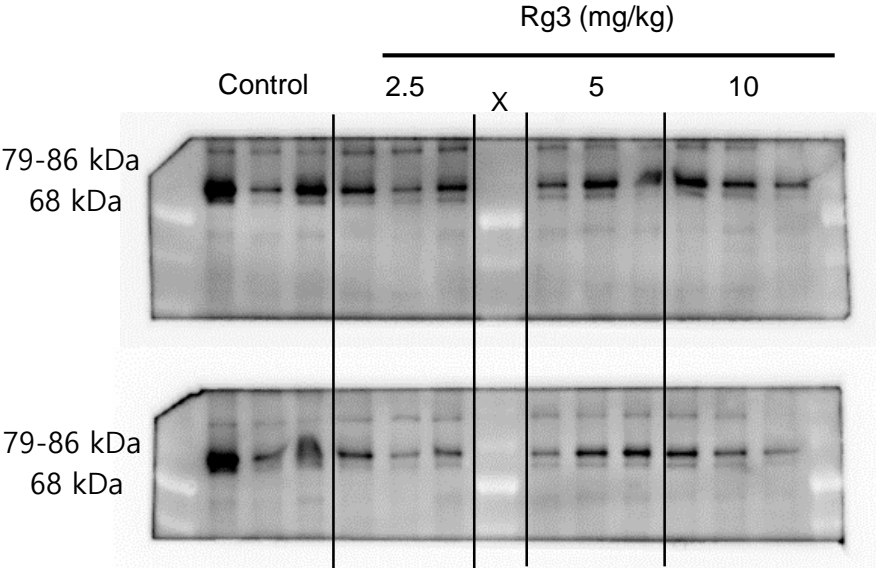

pSTAT3

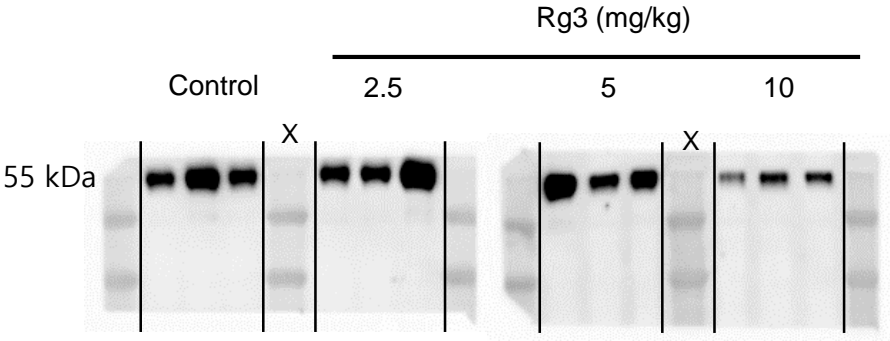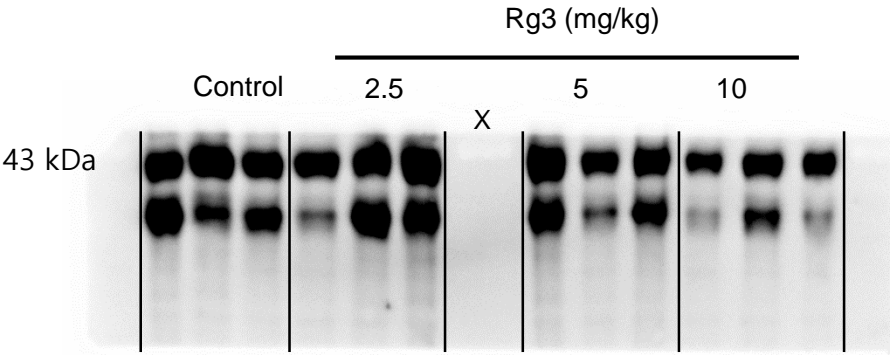

Figure 5D-2

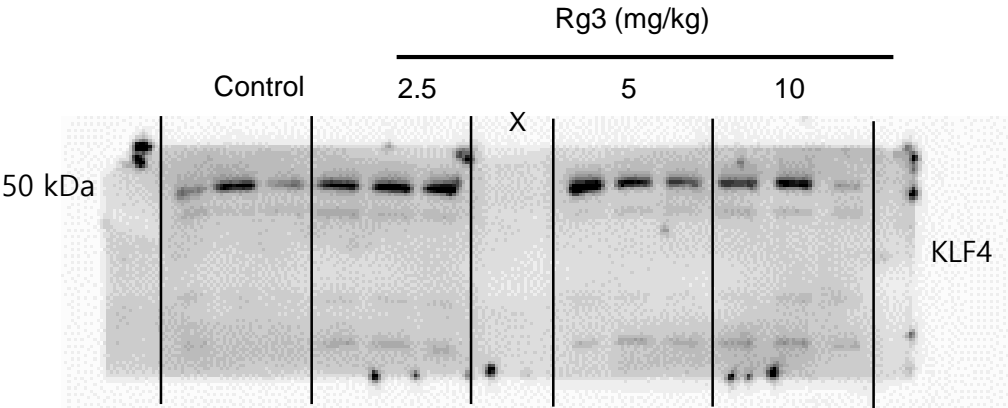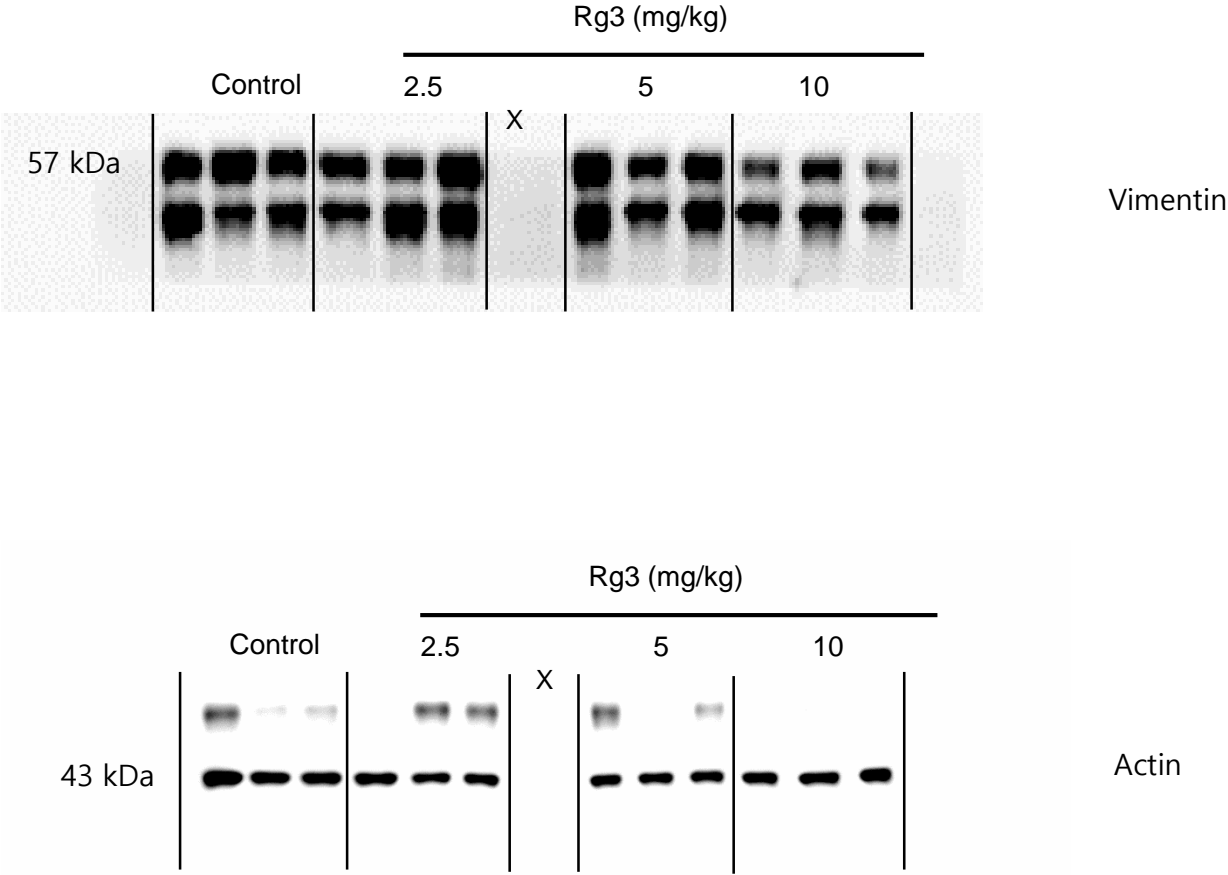

Supplement: S1 Raw images — (PDF) [file pone.0240533.s001.pdf]
